# Using Word Embeddings to Learn a Better Food Ontology

**DOI:** 10.3389/frai.2020.584784

**Published:** 2020-11-26

**Authors:** Jason Youn, Tarini Naravane, Ilias Tagkopoulos

**Affiliations:** ^1^ Department of Computer Science, University of California at Davis, Davis, CA, United States; ^2^ Genome Center, University of California at Davis, Davis, CA, United States; ^3^ Biological Systems Engineering, University of California at Davis, Davis, CA, United States

**Keywords:** food ontology, word embeddings, automated ontology learning, data-driven, machine learning, ontology population, ontology metrics

## Abstract

Food ontologies require significant effort to create and maintain as they involve manual and time-consuming tasks, often with limited alignment to the underlying food science knowledge. We propose a semi-supervised framework for the automated ontology population from an existing ontology scaffold by using word embeddings. Having applied this on the domain of food and subsequent evaluation against an expert-curated ontology, FoodOn, we observe that the food word embeddings capture the latent relationships and characteristics of foods. The resulting ontology, which utilizes word embeddings trained from the Wikipedia corpus, has an improvement of 89.7% in precision when compared to the expert-curated ontology FoodOn (0.34 vs. 0.18, respectively, *p* value = 2.6 × 10^–138^), and it has a 43.6% shorter path distance (hops) between predicted and actual food instances (2.91 vs. 5.16, respectively, *p* value = 4.7 × 10^–84^) when compared to other methods. This work demonstrates how high-dimensional representations of food can be used to populate ontologies and paves the way for learning ontologies that integrate contextual information from a variety of sources and types.

## Introduction

The need for efficient food systems to support food security (Tscharntke et al., 2012; Alexander et al., 2017), food production and distribution (Moe, 1998; Dabbene and Gay, 2011), and nutrition (Lemay et al., 2007; Kretsera et al., 2015; Berners-Lee et al., 2018; Barabási et al., 2019) to serve a growing planet is now more evident than ever (Guyomard et al., 2012). When it comes to food production and composition, various initiatives have proposed data repositories and ontologies regarding ingredients, processes, and final food products. Some examples of food compositional databases are USDA’s FDC (US Department of Agriculture, Agricultural Research Service, 2019) which provides nutrient composition data for approximately 300,000 food entries and FooDB (Wishart, 2018) which provides quantitative chemical composition data in foods covering 80,000 chemicals in 800 foods. Other databases highlight non-ontological aspects, for instance, the GPC database (GS1, 2018) that contains barcodes for food products and the EFSA database (EFSA, 2015), which is a 32-feature categorization system. Concomitantly, there are multiple ontologies in various stages of development and usage (Dooley et al., 2018; Eftimov et al., 2019), with an ontology defined as the body of formally represented knowledge in some area of interest expressed by objects and concepts, and the relationships that hold among them (Genesereth and Nilsson, 2012). A notable example is FoodOn (Dooley et al., 2018), an open-source and formal food ontology curated by the FoodOn consortium, which represents a food item by its properties and adheres to the FAIR standards (Wilkinson et al., 2016). As we move towards a detailed atlas of chemical food composition (Barabási et al., 2019), there is a current and present need for tools and frameworks that are data-driven and automated to support the creation and/or extension of evidence-based, detailed ontologies at scale.

The structure of an ontology is based on the triple of *subject*, *predicate*, and *object* which is similar to that of knowledge graphs (World Wide Web Consortium, 2011), yet there exist subtle distinctions. Ontologies are usually smaller in size, are domain-specific, capture complex relationships between the classes and instances, and can enforce their structure by applying sets of restrictions and rules (Benslimane et al., 2006; Ehrlinger and Wöß, 2016). Moreover, compared to the multi-relational knowledge graphs where different types of predicates can exist, ontologies connect concepts predominantly through subsumption or hypernymy relationships. Nonetheless, due to their structural similarities, several methods developed for the knowledge graph can also be applied to the area of ontology learning which includes tasks ranging from creating ontologies to extending and populating existing ontologies. However, in practice, the choice of embedding depends on the available corpus, and the method is specific to the task at hand. A task commonly seen in knowledge graphs is link prediction, where the starting state is a knowledge graph and the end result is a more accurate and/or more complete knowledge graph. Link prediction uses methods that explain the triples using the latent features such as Poincaré embeddings (Nickel and Kiela, 2017) or extract triples using contextual patterns from some text data. In the area of ontology learning, word embeddings created from text data are used to create and populate an ontology in an one-shot fashion using unsupervised methods such as clustering (Mahmoud et al., 2018) or to populate a skeleton knowledge graph initialized with seed instances in an iterative fashion (Jayawardana et al., 2017; Mitchell, 2018).

**FIGURE 1:**
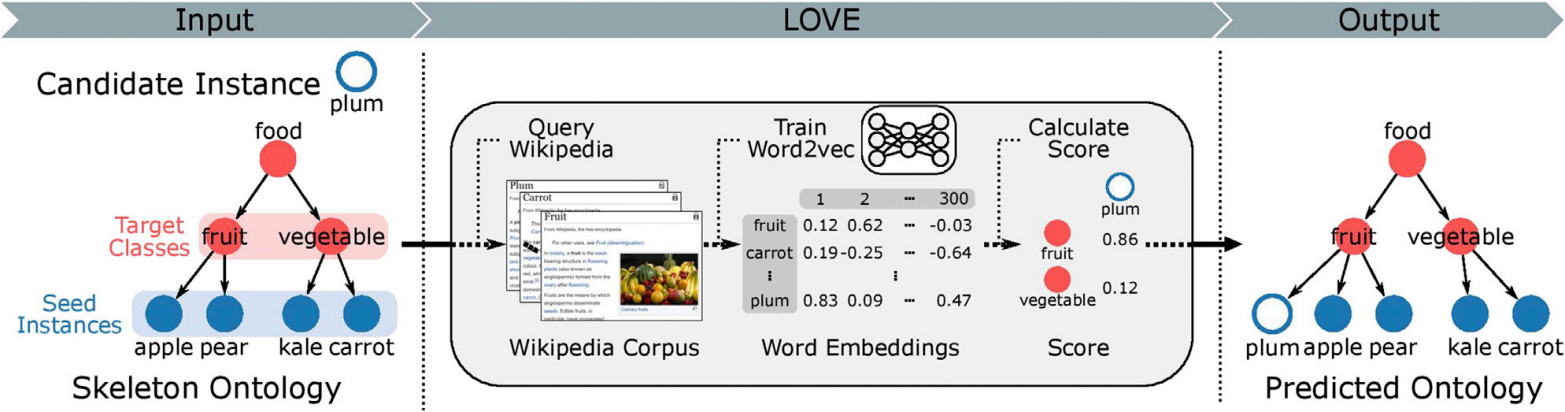
Overview of the LOVE ontology population framework. The hierarchical structure of the ontology is organized as a directed acyclic graph, where a class connects to its parent classes through directed edges. Target class is the parent class of the food instances. Note that some classes are part of the hierarchical ontological structure and do not contain any instances. All class and instance labels are used to query the Wikipedia corpus, which is then used to train food word embeddings. The mapping function then uses the word embeddings to map the candidate instances to the target classes. All relations between the instances and classes are of type “is a.” We compare the predicted ontology to the ground truth ontology and report the performance using precision (more information in the “*Methods*” section).

Here, we address the challenge of how to populate new instances into an existing ontological structure. We introduce LOVE (learning ontologies via embeddings), a semi-supervised framework for the automated ontology population (Figure 1), which uses word embeddings trained on a corpus obtained from Wikipedia. The required memory and computational time of the proposed method scale linearly with increasing number of instances. LOVE was applied on the FoodOn dataset to create the first food ontology using word embeddings. We evaluate the predicted ontology against FoodOn and achieve an increased precision of 89.7% when compared to the best alternate non-embedding-based method that uses Hamming distance (0.34 vs. 0.18, respectively, with a baseline precision of 4.7 × 10^–4^).

## Methods

### Data Preprocessing and Training of Word Embeddings

There are a total of 2,764 classes and 10,865 instances in FoodOn. Every class or food instance is identified by its label. For example, “cow milk cheese” is a class label, and “Brie cheese food product” is a food instance label. These labels are constructed using 4,139 unique words (e.g., “cow,” “milk,” “cheese,” “Brie,” “food,” and “product”). We searched both the labels and their unique constituent words to obtain corresponding Wikipedia pages (Figure 1), which we refer to as Wikipedia corpus. We preprocessed the corpus as follows: lower-case conversion, synonym mapping, punctuation stripping, white-space stripping, numeric stripping, stop-words removal, short words stripping, and lemmatization. Note that the Wikipedia corpus consists of 142,948 unique words. For their training, we used the gensim (Rehurek and Sojka, 2010) implementation of the word2vec skip-gram model (Mikolov et al., 2013). Default settings of the gensim word2vec model were used except for the following parameters: number of epochs of 100, window size of 5, and minimum count of 1. We trained four different dimensions of word embeddings for word2vec: 50, 100, 200, and 300 d. In addition to word2vec, we also tested using the pre-trained word embeddings trained with GloVe (Pennington et al., 2014) and fastText (Joulin et al., 2016; Bojanowski et al., 2017). For GloVe, we downloaded pre-trained word embeddings of dimensions 50, 100, 200, and 300 d known as glove.6B. For fastText word embeddings, we used two different versions of word embeddings of size 300 d that have been trained using different training corpora. Refer to Table 1 for complete information.

**TABLE 1:** Comparison of word similarity methods and their performance.

Method		Training corpus	Average precision	Average distance (hops)	Running time (s)
–	Random	n/a	4.6 × 10^–4^	8.23	65.1
Non-embedding	Jaccard (Niwattanakul et al., 2013)	n/a	0.097	6.45	169.9
	Hamming (Hamming, 1950)		0.181	5.16	111.7
GloVe (Pennington et al., 2014)	50 d	Wikipedia 2014 + Gigaword 5	0.192	4.29	201.8
	100 d		0.228	3.87	
	200 d		0.261	3.53	
	300 d		0.297	3.32	
fastText (Joulin et al., 2016; Bojanowski et al., 2017)	Wikinews	Wikipedia 2017 + UMBC webbase + statmt.org	0.313	2.98	
	Crawl	Common Crawl	0.317	3.00	
Word2vec (Mikolov et al., 2013)	50 d	Subset of Wikipedia 2020	0.262	3.32	
	100 d		0.295	3.09	
	200 d		0.318	2.99	
	300 d		**0.344**	**2.91**	

Average distance denotes average path distance between the predicted class and the ground truth class among 100 multiple randomly selected seed instances. All methods were run parallel on 8 core CPU (16 threads) with 32 GB of memory. Note that the running time excludes the time used for training the word embeddings. Entries in bold correspond to the best performing method.

### Ontology Population

As illustrated in Figure 1, our algorithm aims to map a food instance (e.g., “plum”) through an “is a” relationship to its parent (e.g., “fruit,” ideally), which we refer to as its target class. If we let *i* be a food instance and *c* be a target class, then i∈I and c∈C, where *I* is the group of all food instances we seek to map and *C* is the group of target classes to which we map the food instance. We also define *I*_*c*_ to be all the food instances within a class *c*. To map the instance to its appropriate target class, we propose an approach based on the similarity of word embeddings. Our criteria for optimal population consider a linear combination of two scores:

$$\text{score}\left(c;i\right)=\alpha \cdot {\text{score}}_{\text{siblings}}\left(c;i\right)+\left(1-\alpha \right)\cdot {\text{score}}_{\text{parent}}\left(c;i\right),$$

where *α* controls the ratio of the two terms. score_siblings_ is the similarity of the food instance *i* with the seed instances in *I*_*c*_:

$${\text{score}}_{\text{siblings}}\left(c;i\right)=\text{sim}\left(\vec{\iota },\sum_{i^{\prime }\in I_{c}}\frac{\vec{\iota^{\prime }}}{\left|I_{c}\right|}\right),$$

where $\vec{\cdot }$ is the word embedding vector created by taking the average of the constituent word embeddings, $\left|I_{c}\right|$ is the number of all the seed instances in *I*_*c*_, and sim () is the measure of similarity between the two word embedding vectors. score_parent_ is the similarity of the food instance *i* with the target class *c*:

$${\text{score}}_{\text{parent}}\left(c;i\right)=\text{sim}\left(\vec{\iota },\vec{c}\right).$$

Finally, predicting which target class $\vec{c}$ the food instance *i* will get mapped to can be formulated as follows:

$$\bar{c}=\arg\underset{c}{\max}\text{ score}\left(c;i\right).$$

For the scope of this work, we map the food instance to a single target class even if it was originally mapped to multiple classes. For the case of FoodOn, we observed that the precision of ontology learning increases as the number of seed food instances per class (*n*_seed_) increases (Supplementary Figure S1) as a class is better represented as the number of seed instances increases. For sim (), we used Euclidean distance and cosine similarity, with the latter having better performance and used throughout this work (Supplementary Figure S2). We empirically set *α* = 0.8 after testing all values between 0.0 and 1.0 with an interval of 0.1 (Supplementary Figure S3).

### Evaluation Metrics of the Ontology Structure

The granularity and cohesiveness metrics have to do with fundamental design questions of ontologies such as the optimum number of classes and whether a class is overspecified or underspecified (Whetzel et al., 2011). *Granularity* is semantically defined as the ability to represent different levels of detail in data (Keet, 2008). In our work, we quantitatively define granularity of a certain ontology superclass *c*_*A*_ as

$$\text{granularity}\;\left(c_{A}\right)=\frac{\left|I_{C^{A}}\right|}{\left|C^{A}\right|},$$

where $C^{A}\subseteq C$ is the set of all the classes that have *c*_*A*_ as their superclass, $I_{C^{A}}$ is the set of all food instances belonging to *C* ^*A*^, and *c*_*A*_ is a superclass of *c*_*B*_ if every instance of *c*_*A*_ is also an instance of *c*_*B*_ (Noy and McGuinness, 2011). *Cohesiveness* of a superclass is a measure of subclass semantic relevance, and by corollary, the degree of its subclasses has the same relation to each other (Gangemi et al., 2005). Here, we quantitatively define the cohesiveness of a certain ontology superclass *c*_*A*_ as

$$\text{cohesiveness}\left(c_{A}\right)=\frac{\left|C^{\prime A}\right|}{\left|C^{A}\right|},$$

where *C*′^*A*^ is the set of all correct subclasses within the superclass *c*_*A*_. For example, in the superclass “cheese food product by organism” in FoodOn, the subclasses “cow cheese,” “goat cheese,” “sheep cheese,” and “buffalo milk cheese” are correct, while the subclass “blue cheese” is not since it describes a method/process and not the point of origin. In this case, the cohesiveness value would be 4/5 = 0.8. Another example is in the case of the bean superclass where the subclasses that are bean varieties are correct and subclasses for processed forms of beans such as “bean flour” are not. The cohesiveness of the cheese superclass is 0.52, implying that only half of the subclasses are correct, and the bean superclass has a higher cohesiveness of 0.93 (Supplementary File 2).

### Success Metric of the Ontology Population

We use precision to assess the performance of the ontology population and define it as follows:

$$\text{precision}=\frac{\text{TP}}{\left(\text{TP}+\text{FP}\right)},$$

where a food instance $i\in I_{c}$ is considered a TP if and only if the mapping function correctly placed *i* under *c*, and FP, otherwise. In addition, we define the path distance to be the shortest distance (hops) between the predicted class and the ground truth class, where a perfect ontology population algorithm would have a path distance of 0.

## Results

### Structural Topology of FoodOn

**FIGURE 2:**
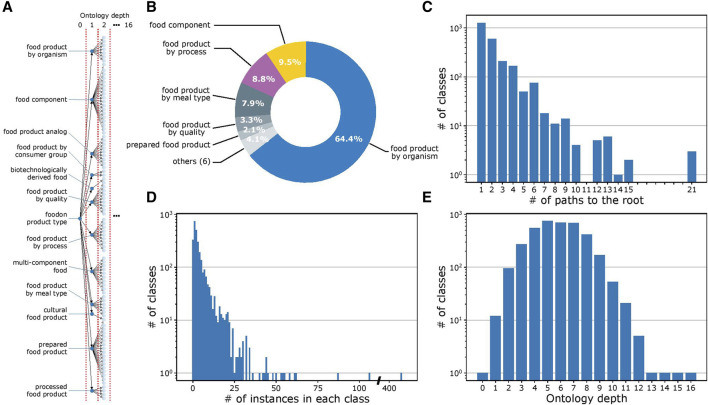
FoodOn structure analysis. **(A)** Visualization of the partial FoodOn class hierarchy. FoodOn contains 10,865 food instances (not shown here) that are mapped to one or more of the 2,764 classes. All classes in FoodOn branch out from a single root class "foodon product type" located at depth 0. **(B)** Pie chart showing the proportion of subclasses for each of the 12 classes in the highest, i.e., the first, level of the ontology. Each of these classes represents one of the 12 features of a food item. **(C)** Histogram showing the number of paths to the root class for each of the 2,764 classes. Number of paths considers the multi-parent architecture. **(D)** Histogram showing the number of instances in each class. Only 2,433 of 2,764 classes have instances. Certain classes only have subclasses aimed at providing further levels of differentiation. The vast range in instances per class indicates that specialized classes with fewer instances are more typical to the ontology, though there are some classes with up to 100s of instances. **(E)** Histogram showing the number of the target classes at the respective ontology depth. This representation defines the ontology depth of the class as the number of intermediate classes in a path connecting it to the root class.

Figure 2A provides a visualization of the ontology structure for the 2,764 classes in FoodOn. At the highest level of the ontology, every food item is described by various features, which minimally include its source organism and up to 11 other features, with each feature represented as a class (processes and material quality, among others; Figure 2B). A complete examination indicates that the ontology structure is heterogeneous in its granularity, with some classes having many subclasses and interconnectivity, while others have only one subclass. In a similar trend, while some classes have hundreds of instances, other classes have only one (Figures 2C and D). Figure 2E illustrates the variation in ontology depth for a given class, which is defined as the number of intermediate classes present in a given path that connects it to the root (Blanchard et al., 2005). Considering all the factors mentioned above, the FoodOn ontology is highly granular with an average of 3.15 food instances per class.

### Granularity and Cohesiveness Impair Precision of Automated Methods

**FIGURE 3:**
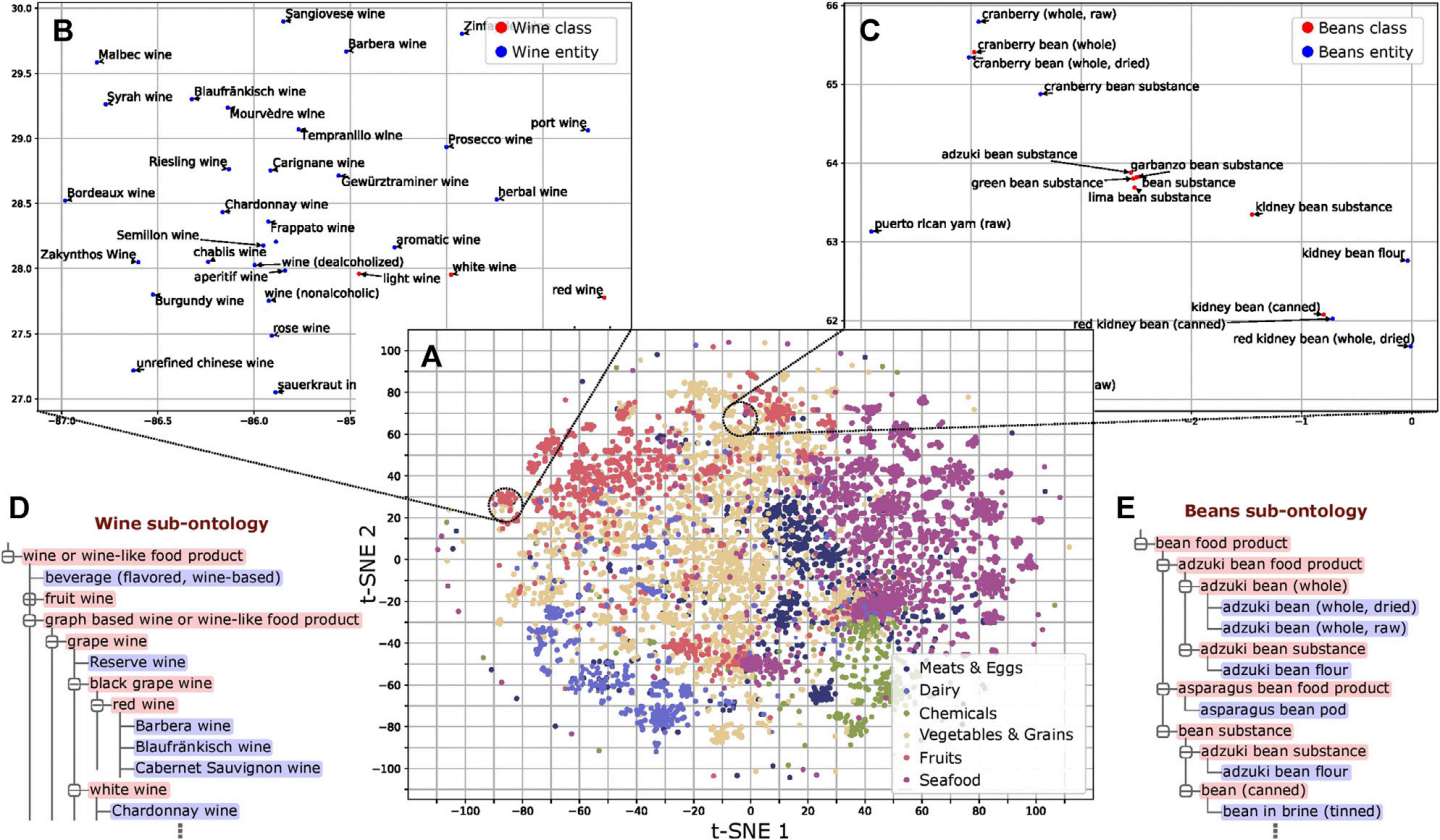
Analysis of word embeddings. **(A)** t-SNE plot of the FoodOn class and instance labels based on the word embeddings. The distribution pattern of the embeddings shows ordering consistent with that of the FoodOn hierarchy (*p* value < 0.0001). **(B)** Wine subsection follows uniform spatial distribution of instances and classes. **(C)** Bean subsection shows regional crowding of instances/classes due to the repetitive words in the label. **(D,E)** Wine and bean related sub-ontologies as found in FoodOn, with the bean being significantly more granular (more classes) than expected. Classes and food instances are highlighted red and blue, respectively.

We trained word embeddings for the 13,629 instances and class labels in FoodOn to use in our method. These embeddings capture latent information of the food type as revealed by dimensionality reduction (Maaten and Hinton, 2008) and subsequent analysis (Figure 3A and Supplementary File 1). Regarding the structure of FoodOn, the granularity differs substantially as shown in Figures 3B–E, where we compare the superclasses “wine” and “beans,” with granularity 5.64 vs. 1.96, respectively. We also noticed inconsistencies in the further classification of each superclass which we quantify by the cohesiveness. Relevant to our work of ontology learning, we found that both the cohesiveness and the granularity are positively associated with better ontology population performance (PCC of 0.56 and 0.51, respectively; *p* value = 2.5 × 10^–2^ and 4.5 × 10^–2^, respectively) (Supplementary Figure 4; Supplementary File 2).

### Learning Ontology via Embeddings Leads to Substantially Better Performance

**FIGURE 4:**
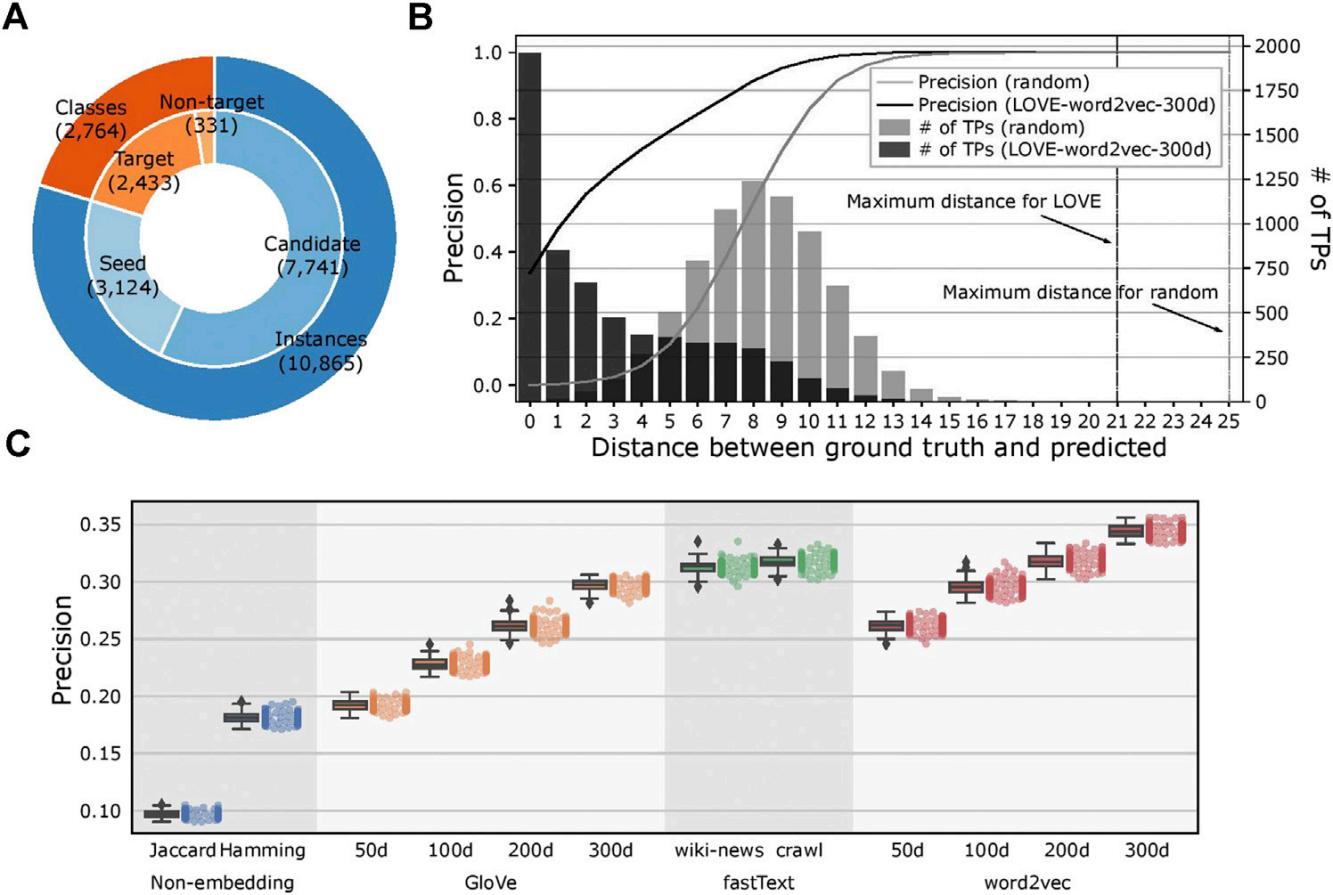
Evaluation of the LOVE framework on a food ontology. **(A)** Number of ontology classes and food instances that were used for the LOVE-derived ontologies. Candidate instances are mapped to one of the target classes by LOVE, and each target class is initialized by seed instances. Classes without instances are not considered as target classes. **(B)** Distribution of precision and number of true positives of the mapped ontology as a function of shortest distance (hops) between the predicted class and the ground truth class for LOVE (black) and random assignment (gray) (*p* value = 4.8 × 10^−102^). **(C)** Precision of the ontology population for different similarity methods.

We kept the ontological structure of FoodOn unchanged with 2,433 target ontology classes and created 100 different seeded-skeleton ontologies to test the statistical significance of the methods by selecting two random seeds for each target class. This process resulted in 3,124 food instances used as seeds from a total of 10,865 instances, and the task was to map the remaining 7,741 food instances to the target classes (Figure 4A; Supplementary File 3). The LOVE-generated ontology, which uses the word embeddings of size 300 d trained using the Wikipedia corpus, had a significantly reduced path distance from what is expected from random chance (*p* value = 4.8×10^–102^; Figure 4B). Moreover, ontology population methods based on the word embeddings performed better when compared to the traditional text similarity methods regardless of the embedding size or the training algorithm, with an 89.7% increased precision (0.34 vs. 0.18, respectively, *p* value = 2.6×10^–138^) and a 43.6% shorter path distance (2.91 vs. 5.16, respectively, *p* value= 4.7×10^–84^; Figure 4C; Table 1).

## Discussion

As shown in Figure 3A, there is an alignment of the word embeddings and the FoodOn classes at a high level. However, through deeper analysis of the ontology structure and the results of automated ontology learning, we discovered the causes for discrepancies between the user-defined ontology and the ontology representation from the corpus. The granularity and cohesiveness issues impacting the precision have to do with a well-known and fundamental design question of how many classes are too few or too many (Noy and McGuinness, 2011). The classes with lower than average granularity of 4 combine several features of a food item such as its source, process, and organoleptic quality. However, the nomenclature is not consistent as it varies from a long and precise class name to less-precise representations. This is not a scalable approach to a data-driven automated ontology since it will require manually curated classes when mapping foods of yet unknown features such as sources and processes. Moreover, it will lead to errors in mapping class-class and class-instance relations if done manually, as the ontology grows. To avoid these issues, an extension would be for every variety-specific subclass to contain a flat list of instances. For example, in Figure 3E, the food instance “adzuki bean flour” is mapped to two parent classes in the bean superclass. Instead, the “product by process” class at a depth of one can have a subclass of “milled food” which aggregates all the flour variants and notably the “bean flour” class. This also addresses the problem of cohesiveness described in the “*Methods*” section. The ontology learning function can then be applied on each of the 12 highest parent classes (Figure 2B).

Taking into account the structural similarity between the ontology and the knowledge graph, we considered applying observable and latent feature-based link prediction models (Toutanova et al., 2015; Grover and Leskovec, 2016; Lao and Cohen, 2010) to populate the ontology. However, such models either are dependent on external data or require at least one pre-existing path connecting the candidate instance to the target class. A possible extension to our work is to train the word embeddings using other related corpora such as food-related literature and databases, for example, the FDC database (US Department of Agriculture, Agricultural Research Service, 2019). Moreover, the pertinent information can be extended to chemical composition, phenotypic effects, and association with health states. Another natural extension would be to train methods that encode the hierarchical structure of the knowledge graphs, such as Poincaré embeddings (Nickel and Kiela, 2017), with hierarchical food domain data (Haussmann et al., 2019) for the ontology population task. Along with an optimally designed skeleton ontology, we expect that these improvements would lead to much improved accuracy of the automatically generated ontology.

## Conclusion

In this work, we applied the learning ontologies via embeddings (LOVE) framework, which takes advantage of the semantic similarity of the word embeddings to the field of food ontologies. The automated method we proposed here is a solution to the manual burden of populating an ontology with continuous influx of new data. Therefore, the desired automation would be a semi-supervised method that yields high precision, with minimal manual intervention. Although the importance of automated ontology learning has been discussed before (Drumond and Girardi, 2008), to the best of our knowledge, this is the first time a solution is applied to an existing ontology in the food domain. We believe that our work is a step towards the fully automated ontologies.

## Data Availability Statement

All data, code and instructions on how to reproduce the results are available at https://github.com/IBPA/LOVE.

## Author Contributions

TN and JY contributed equally in preparation for the manuscript.

## Conflict of Interest

The authors declare that the research was conducted in the absence of any commercial or financial relationships that could be construed as a potential conflict of interest.
