# Supplementary material for: Using Word Embeddings to Learn a Better Food Ontology
Source: Front Artif Intell. 2020 Nov 26;3:584784. doi: 10.3389/frai.2020.584784 (PMC7861243; doi:10.3389/frai.2020.584784)
Supplement: Supplementary file 4 [file datasheet4.pdf]

# Using Word Embeddings to Learn a Better Food Ontology

Jason Youn[1,2], Tarini Naravane[2,3], and Ilias Tagkopoulos[1,2]*

[1]Department of Computer Science, University of California at Davis
[2]Genome Center, University of California at Davis
[3]Biological Systems Engineering, University of California at Davis
*itagkopoulos@ucdavis.edu

## SUPPLEMENTARY FIGURES

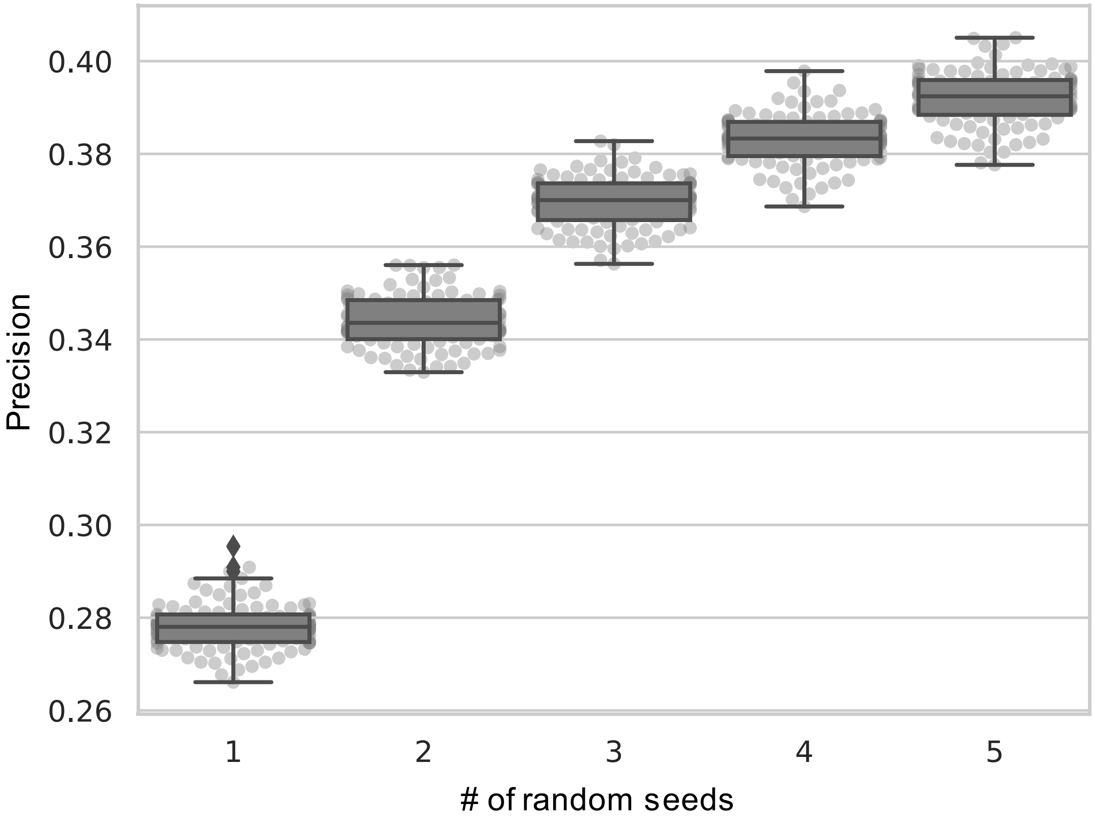


**Supplementary Figure 1. Precision with respect to the number of random seeds used.** Precision of FoodOn ontology mapping increases as the number of random seeds for each target class increases. In the case of FoodOn, we arbitrarily set $n_{seed} = 2$.

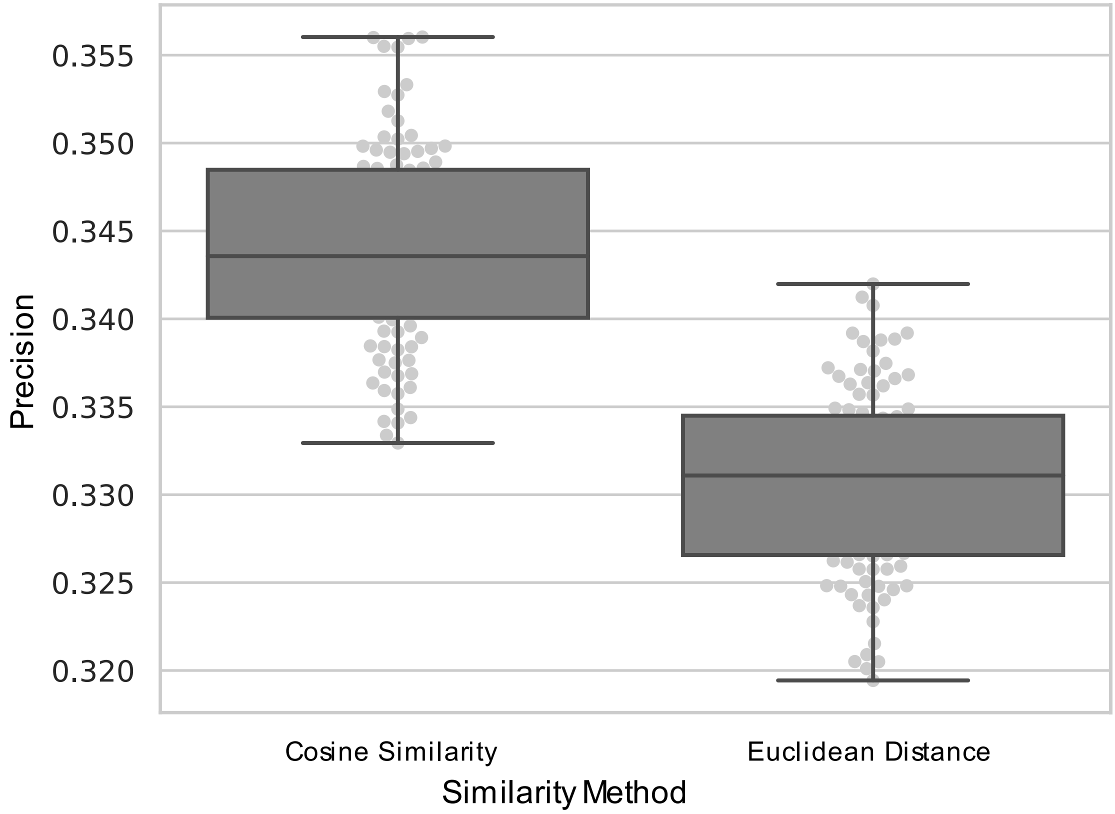


**Supplementary Figure 2. Comparison of cosine similarity and Euclidean distance.** For measuring similarity between the word embeddings, cosine similarity had better performance than the Euclidean distance (0.34 vs. 0.33, precision respectively; *p*-value = 8.1 $10^{-54}$).

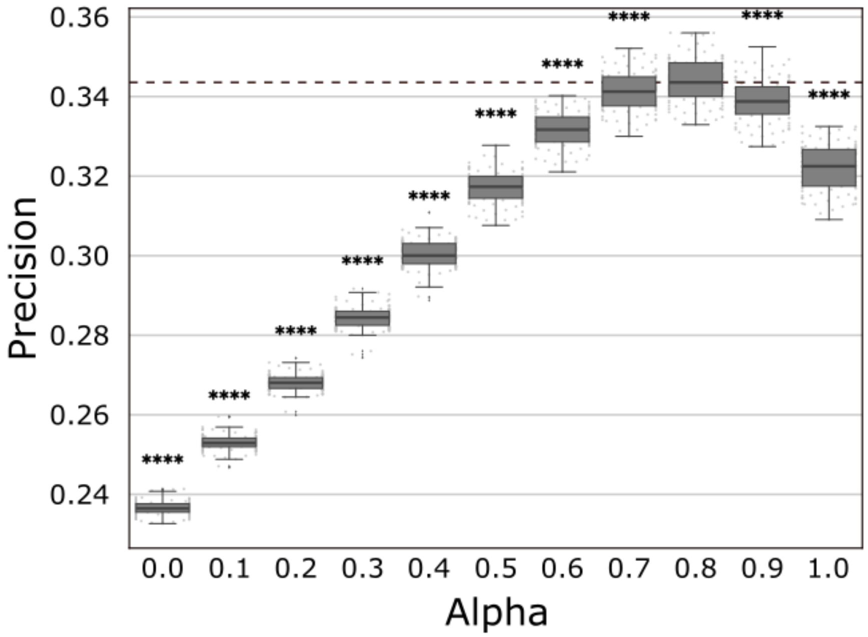


**Supplementary Figure 3. Precision of ontology mapping with respect to the hyperparameter alpha.** Alpha controls the balance between the similarity of candidate entity with the target class or seed entities of the target class. The best performance is for alpha = 0.8 and the grey dots denote samples.

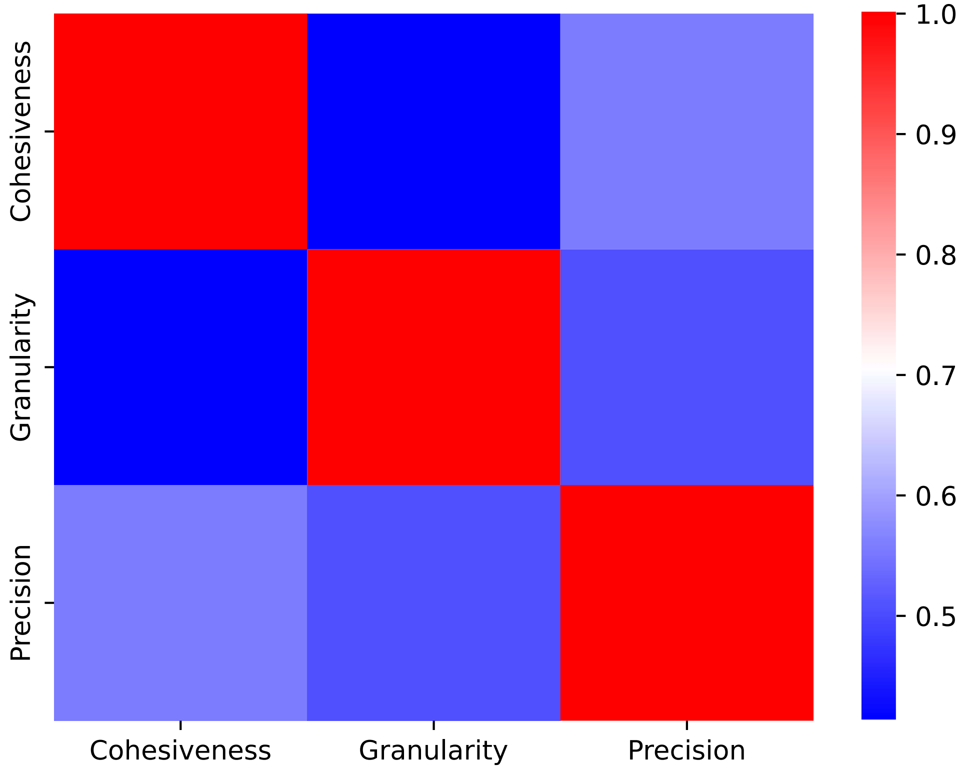


**Supplementary Figure 4. Pairwise Pearson correlation between the artifacts of an ontology and the precision of ontology mapping.** Both cohesiveness and granularity are positively correlated with the precision of ontology mapping (PCC of 0.56 and 0.51, respectively; *p*-value = 2.5 $10^{-2}$ and 4.5 $10^{-2}$, respectively). Albeit not statistically significant, there also exist a positive correlation between the cohesiveness and granularity (PCC of 0.41, *p*-value = 1.1 $10^{-1}$).
